# Supplementary material for: Decoding Sound and Imagery Content in Early Visual Cortex
Source: Curr Biol. 2014 Jun 2;24(11):1256–62. doi: 10.1016/j.cub.2014.04.020 (PMC4046224; doi:10.1016/j.cub.2014.04.020)
Supplement: Document S1. Supplemental Experimental Procedures, Figures S1–S3, and Table S1 [file mmc1.pdf]

Current Biology, Volume *24*

Supplemental Information

Decoding Sound and Imagery Content

in Early Visual Cortex

Petra Vetter, Fraser W. Smith, and Lars Muckli

Supplemental Material

Supplemental Results

Experiments 1 - 4

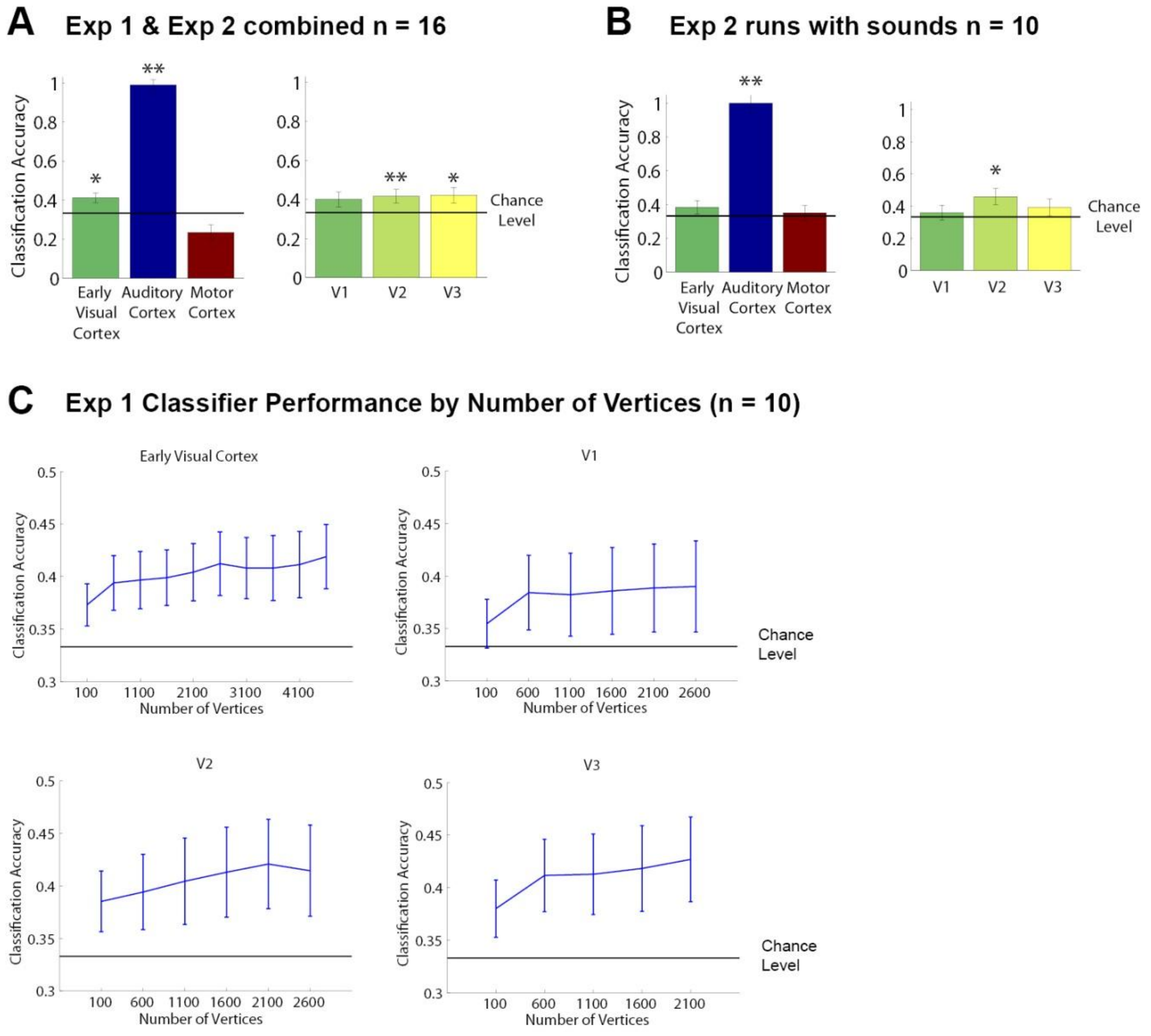

Figure S1. Classification results from Exp. 1 & 2. A) Results from the pooled data set with higher statistical power (n = 16). Here the data of Exp. 1 was pooled with the data of the runs with sound stimulation in Exp. 2, excluding the subjects who participated in both experiments (n = 4). B) Results from Exp. 2 for the runs with sound stimulation, replicating the results of Exp. 1. C) Classification performance in Exp. 1 as a function of number of vertices in each visual ROI. All error bars indicate SEM.

Effect sizes ((mean decoding accuracy-chance)/ STD) for the pooled data set were as follows: Early visual cortex: 0.79; V2: 0.60; V3: 0.57; Auditory cortex: 23.0.

Comparison of confusion matrices between Exp. 1 and Exp. 3 for V3 and early visual cortex revealed no significant difference (repeated measures ANOVA, $p = .60$) and a significant correlation in early visual cortex (Spearman's $r = .726$; $p = .027$). That is, successful sound decoding in both experiments relied on similar patterns of classifier performance.

Repeated measures ANOVA for classification accuracies across the early visual areas V1, V2 and V3 revealed non-significant effects of visual area ($F(2,18) < 1.7$, $p > .20$) in all experiments. Therefore, the data does not allow us to conclude a differential involvement of individual early visual areas across the different experiments.

Behavioural task performance (mean accuracy) in Exp. 3 & Exp. 4:

Exp. 3: 90.4% (SEM 2.9); Exp. 4: 90% (SEM .02).

Whole-brain Searchlight Results

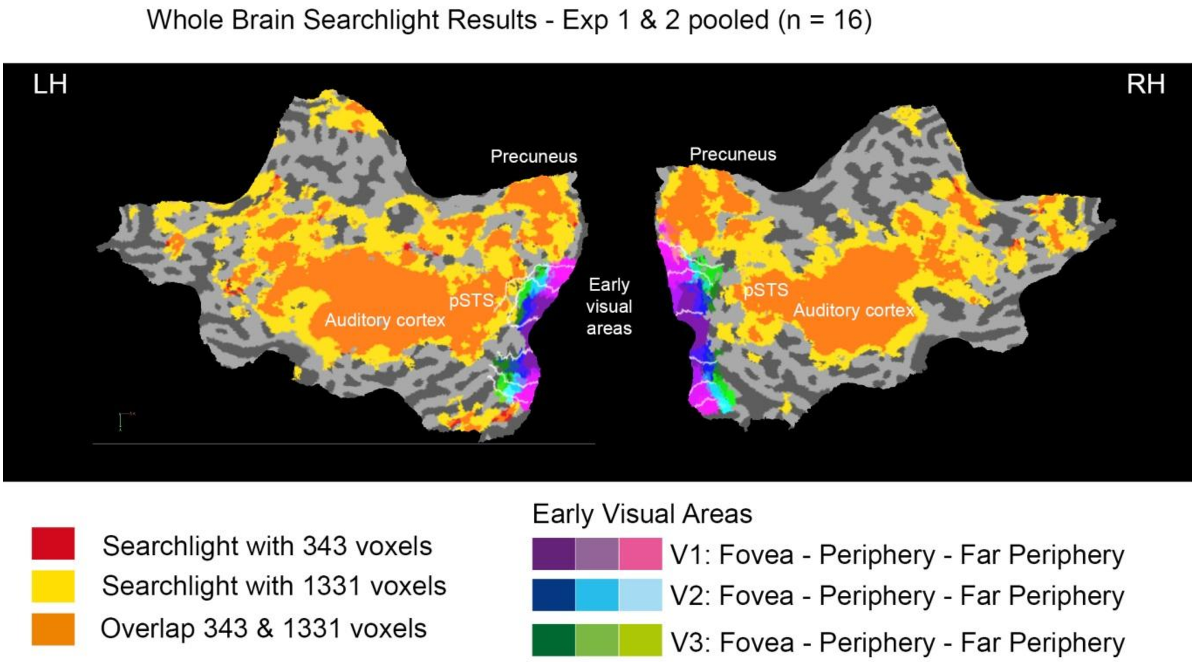

Figure S2. Searchlight results for the pooled data set (Exp. 1 & 2, n = 16) for two different searchlight sizes (a cube of 343 voxels and a cube of 1331 voxels).

Univariate Results

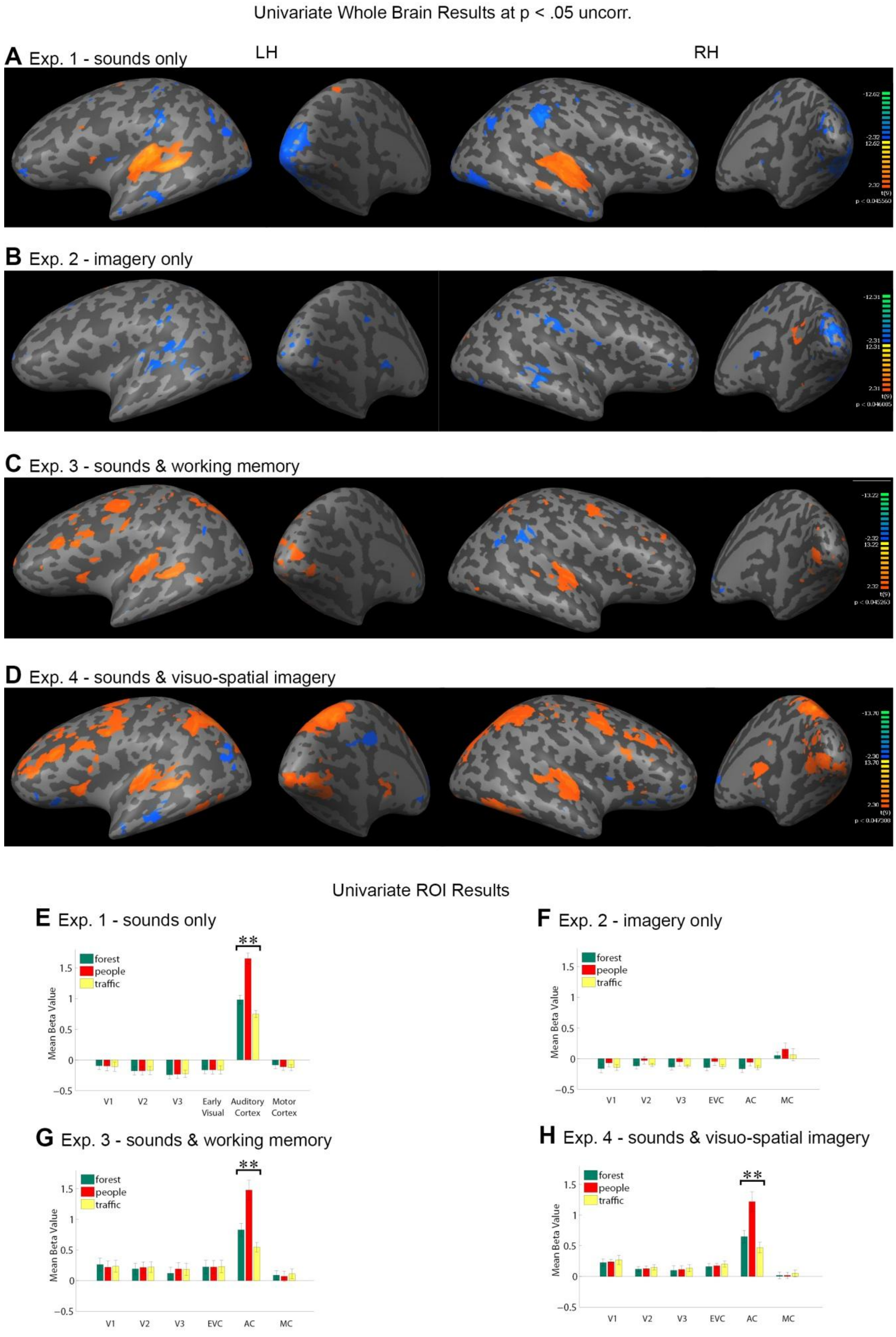

Fig. S3. Results of the univariate analysis for Exp. 1- 4.

A) - D) Whole-brain results from a random effects analysis projected onto cortical surfaces (MNI template) for Exp. 1- 4 at $p < .05$ uncorr. E) - H) Mean beta values for the three heard or imagined sounds in the relevant ROIs. Error bars indicate SEM. ** indicate $p <.001$ for a differential effect of sounds.

Sound display activated auditory cortex in Exp. 1, 3 & 4 and the orthogonal tasks of Exp. 3 and 4 activated a network of parietal and frontal regions due to the task demands (stronger activity for Exp. 4, particularly in parietal regions typically related to visuo-spatial processing). Note that even at very liberal thresholds of $p >.05$ (uncorrected), no positive activation was found in early visual areas, instead weak deactivation in Exp. 1 and 2.

Classification Results of Experiment 5

All classifications between categories and all cross-classifications were highly successful in auditory cortex (p =. 001).

	**Traffic 1**	**Traffic 2**	**Airplane**
**People 1**	V2 & V3* (.542 /.021)	--	V2* (.532 / .019)
**People 2**	V1 *** (.553 / .020)<br>EVC* (.532 / .026)	V3d * (.543 / .032)<br>V2&V3* (.535 / .030)	V1* (.537 / .017)<br>V3v* (.547 / .020)<br>EVC** (.565 / .023)
**Kids**	V3 *** (.555 / .017)	V1* (.550 / .026)<br>V2* (.528 / .037)<br>V3*** (.578 / .041)<br>V2&V3* (.538 / .040)<br>EVC** (.550 / .032)	V3d*** (.560 / .020)<br>V3** (.558 / .032)

**People 1 vs Traffic 1 X People 2 vs Traffic 2**	V2&V3* (.524 / .014)
**People 2 vs Traffic 2 X Kids vs Airplane**	V2* (.531 / .016)<br><br>V2&V3* (.523 / .013)
**People 1 vs Traffic 1 X Kids vs Airplane**	--

Supplemental Table 1. Classification Accuracies of Exp. 5.

Early visual areas with significant above chance classification for all combinations of "human" versus "inanimate" sounds, and cross-classification between one pair of exemplars against another, shown with mean classification accuracy and SEM (acc / SEM). All p - values were derived from permutation analyses. *p < . 05, ** p < .005, *** p = .001.

In theory, there is the possibility that differential eye movements in response to the sounds might have caused the differential activity patterns in early visual areas. We have no indication to believe that subjects moved their eyes systematically while being blindfolded and even if they did, activity patterns should not have been caused by retinal stimulation. Furthermore, two pieces of evidence in our findings speak against this possibility. First, the searchlight analysis did not yield successful classification in frontal eye fields. Second, Exp. 5 showed that sound decoding is based on categorical information, and it is not plausible to assume that eye movement patterns should follow this categorical distinction.

Supplemental Experimental Procedures

Subjects, stimuli and experimental design

10 healthy subjects with normal hearing and vision were scanned in each of the five experiments. Subjects signed informed consent. The study was approved by the ethics committee of the College of Science and Engineering, University of Glasgow. Stimulation was solely auditory through noise-reducing headphones (Nordic NeuroLab); subjects wore a blindfold, were instructed to keep their eyes closed at all times and room lights were switched off. The three natural sound stimuli used in Exp. 1- 4 consisted of one exemplar each of traffic noise (a busy road with cars and motorbikes), a forest scene (birds singing and a stream) and a crowd scene (people talking without clear semantic information) and were downloaded from www.soundsnap.com and cut to 12s. In Exp. 5, in addition to the traffic and people sound from experiments 1, 3 & 4, sounds of another traffic scene, a starting airplane, another crowd scene and playing children were used. Here, sounds were cut to 6s. In all experiments, sounds were normalised for amplitude and presented mono.

Natural sounds (Exp. 1, 3, 4 & 5) or a 12s imagery period (Exp. 2) were repeated 6 times per run (pseudo-randomised, but never repeating two of the same after the other). For timings and experimental design, see Fig. 2 & 3. In Exp. 3 & 4, subjects indicated match or mismatch with a right hand button press on a response pad. 4 runs (222 volumes each) were recorded in Exp. 1-4, 5 runs (225 volumes each) in Exp. 5. In Experiment 2, a replication of Experiment 1 was incorporated such that runs with real sound stimulation alternated with runs with imagery cues (4 runs of each type, 8 runs in total).

fMRI data acquisition and analysis

Blood oxygen level dependent signals were acquired in a 3 T Siemens Tim Trio (TR = 2s, TE = 30 ms, resolution 2.5 x 2.5 x 2.5 mm, 35 slices, flip angle 77º, iPAT factor 2). Early visual areas were identified in each individual subject using standard retinotopic polar mapping [13; S1-S2]. In Exp. 1, 3, 4 & 5, auditory cortex was identified as the area in superior temporal sulcus with peak activation for the contrast Sound Stimulation > Baseline. In Exp. 2, auditory cortex was identified from the interleaved runs with sound stimulation. In Exp. 3 & 4, motor cortex was defined in only the left hemisphere as the peak activation for the contrast Right Hand Button Press > Baseline. In Exp. 1 & 2, motor cortex was defined by overlaying the averaged group-level peak activation of Exp. 3 or 4 onto the individual brains of those subjects who did not participate in either Exp. 3 or 4. Data were analysed with BrainVoyager QX (BrainInnovation) with standard preprocessing (including slice scan time correction, no spatial smoothing, temporal high-pass filter, 3D rigid body motion correction). Regions of interest (ROIs) were defined on individual reconstructed cortical surfaces and based on retinotopic mapping. Single block beta weights were estimated for all vertices of each ROI during natural sound stimulation or imagery period [38] and fed into a linear support vector machine classification algorithm (LIBSVM toolbox [S3]). Beta values were normalised in the training data set and the same normalisation was applied for the testing data. The classification was performed one-versus-one for each of the three combinations of sounds and results were averaged. ROIs were combined across both hemispheres, whereas for motor cortex, only the activity patterns of the left hemisphere were analysed (due to right-hand button press). Mean number of vertices across all subjects (combined hemispheres) were as follows: V1: 4908 (SEM 245), V2: 3503 (SEM 164), V3: 2792 (SEM 126),

all early visual cortex: 11236 (SEM 429), auditory cortex ROI: 3311 (SEM 346), motor cortex ROI: 535 (SEM 72)). In Exp. 1-4, the classifier was trained on 3 runs to distinguish between the three types of natural sounds and tested on the remaining 4th run in a leave-one-run-out cross-validation procedure (results were averaged across different folds of training and test data set assignments). For the cross-classification analysis of Exp. 2, the classifier was trained on the runs with sound stimulation and tested on the runs with imagery cues and vice versa, and the results averaged. In Exp. 5, the classifier was trained on 4 runs to distinguish between the two sound categories (human and inanimate) and tested on the remaining 5th run in the same cross-validation procedure. Here, the classification was performed for all 9 combinations of inanimate versus human sound exemplars and the cross-classification was performed for the 3 combinations of one pair of exemplars versus one of the other two pairs. Within each cross-classification, results from training one data set and testing the other and vice versa were averaged. To determine statistical significance, a permutation analysis was performed for all experiments and all classifications, providing a more robust test of statistical significance than a one-sample t-test against chance [S4]. Here, the classifier was trained and tested across 1000 permutations with randomised labels in each subject and each ROI. P values were derived as the probability of getting a value as large as the real label performance in the randomisation distribution, resulting in a smallest possible p-value of 0.001 [S5]. On the group level, p-values were derived from the mean randomisation distribution and the mean real label performance. In Exp. 1-4, p-values were corrected for multiple comparisons with a single threshold test [S5] for the individual visual areas V1, V2 and V3.

Whole brain searchlight analyses were performed on the voxel level with the

SearchMight toolbox [S6] using a linear SVM (with C=1). Each searchlight consisted of 343 voxels (a cube with 7 voxels length, equal to 2744 cubic mm). Statistical significance was assessed by testing whether the mean accuracy across participants was significantly higher than chance (1/3) at each voxel (see also [S7]). Results were corrected for multiple comparisons with a cluster threshold correction ($p < .05$) estimated by the BrainVoyager Cluster Threshold Plugin tool.

Supplemental References

S1. Wandell, B. A., Dumoulin, S. O., & Brewer, A. A. (2007).Visual field maps in human cortex. Neuron, 56(2), 366–383.

S2. Schira, M. M., Tyler, C. W., Breakspear, M., & Spehar, B. (2009). The foveal confluence in human visual cortex. J. Neurosci, 29(28), 9050–9058.

S3. Chang, C.C., Lin, C.J. (2001). http://www.csie.ntu.edu.tw/~cjlin/libsvm.

S4. Stelzer, J., Chen, Y., & Turner, R. (2013). Statistical inference and multiple testing correction in classification-based multi-voxel pattern analysis (MVPA): random permutations and cluster size control. NeuroImage, 65, 69–82.

S5. Nichols, T.E. & Holmes, A.P. (2002). Nonparametric permutation tests for functional neuroimaging: a primer with examples. Hum Brain Mapp, 15, 1-25.

S6. Pereira, F., & Botvinick, M. (2011). Information mapping with pattern classifiers: a comparative study. NeuroImage, 56 (2), 476–496.

S7. Walther, D. B., Caddigan, E., Fei-Fei, L., & Beck, D. M. (2009). Natural scene categories revealed in distributed patterns of activity in the human brain. J. Neurosci, 29(34), 10573–10581.
