# Supplementary material for: Decoding Sound and Imagery Content in Early Visual Cortex
Source: Curr Biol. 2014 Jun 2;24(11):1256–62. doi: 10.1016/j.cub.2014.04.020 (PMC4046224; doi:10.1016/j.cub.2014.04.020)
Supplement: Document S2. Article plus Supplemental Information [file mmc2.pdf]

Current Biology 24, 1256–1262, June 2, 2014 ©2014 The Authors http://dx.doi.org/10.1016/j.cub.2014.04.020

Report

Decoding Sound and Imagery Content in Early Visual Cortex

Petra Vetter,[1,2,*] Fraser W. Smith,[1,3] and Lars Muckli[1,*]
[1]Centre for Cognitive Neuroimaging, Institute of Neuroscience and Psychology, College of Medical, Veterinary and Life Sciences, University of Glasgow, 58 Hillhead Street, Glasgow G12 8QB, UK
[2]Laboratory for Behavioral Neurology and Imaging of Cognition, Department of Neuroscience, Medical School and Swiss Center for Affective Sciences, University of Geneva, Campus Biotech, Case Postale 60, 1211 Geneva, Switzerland

Summary

Human early visual cortex was traditionally thought to process simple visual features such as orientation, contrast, and spatial frequency via feedforward input from the lateral geniculate nucleus (e.g., [1]). However, the role of nonretinal influence on early visual cortex is so far insufficiently investigated despite much evidence that feedback connections greatly outnumber feedforward connections [2–5]. Here, we explored in five fMRI experiments how information originating from audition and imagery affects the brain activity patterns in early visual cortex in the absence of any feedforward visual stimulation. We show that category-specific information from both complex natural sounds and imagery can be read out from early visual cortex activity in blindfolded participants. The coding of nonretinal information in the activity patterns of early visual cortex is common across actual auditory perception and imagery and may be mediated by higher-level multisensory areas. Furthermore, this coding is robust to mild manipulations of attention and working memory but affected by orthogonal, cognitively demanding visuospatial processing. Crucially, the information fed down to early visual cortex is category specific and generalizes to sound exemplars of the same category, providing evidence for abstract information feedback rather than precise pictorial feedback. Our results suggest that early visual cortex receives nonretinal input from other brain areas when it is generated by auditory perception and/or imagery, and this input carries common abstract information. Our findings are compatible with feedback of predictive information to the earliest visual input level (e.g., [6]), in line with predictive coding models [7–10].

Results

Decoding of Sound and Imagery Content in Early Visual Cortex

We used fMRI in combination with multivariate pattern analysis (MVPA) to explore how complex information from audition and imagery translates to the coding space of early visual cortex in the absence of feedforward visual stimulation. Throughout our experiments, we omitted any visual stimulation by blindfolding our subjects (Figure 1). In experiment 1, subjects listened to three types of natural sounds: bird singing, traffic noise, and a talking crowd (see Figure 2). fMRI activity patterns were extracted from retinotopically mapped visual areas 1, 2, and 3 (V1, V2, and V3) (Figure 1 [11]) and fed into a multivariate pattern classifier (linear support vector machine; see Supplemental Experimental Procedures available online). The classifier successfully discriminated the three different sounds in early visual cortex, particularly in V2 and V3 (at ~42%; see Figure 2; for results with increased statistical power, see Figure S1A). Hence, activity patterns in early visual cortex contained sufficient information from auditory stimulation to allow the content-specific discrimination of natural sounds. As expected, the classifier performed very well in auditory cortex (positive control), but not in an unrelated cortical area (motor cortex; negative control). At different eccentricities, classification was successful in peripheral and far peripheral areas, particularly in V1 and V2, but not in foveal regions, consistent with structural and functional evidence for auditory influences on early visual cortex (e.g., [12–14]).

Sounds could have induced crossmodal top-down expectations or mental imagery, which can be conceptualized as one form of nonretinal input to early visual cortex. In experiment 2, we investigated whether sounds could be decoded in early visual cortex even when they were merely imagined and whether feedback information from real and imagined sounds elicited similar activity patterns. Here, runs with natural sound stimulation were interleaved with runs in which subjects solely imagined the sounds upon hearing the word cues "forest," "traffic," or "people" (Figure 2D). Subjects were instructed to engage in mental imagery of the sounds and a corresponding natural scene. Successful discrimination of imagined sounds was observed in both foveal and peripheral areas of early visual cortex (but not far periphery), in V1 and auditory cortex (Figures 2E and 2F; classification of real sounds replicated the results of experiment 1, cf. Figure S1B). Therefore, even in the absence of both visual and auditory stimulation, the contents of mental imagery could be decoded from both V1 and auditory cortex.

Furthermore, we performed a cross-classification analysis between auditory perception and imagery, i.e., we trained the classifier on runs with sound stimulation and tested on runs with pure imagery and vice versa. Cross-classification succeeded in V1 and V2 (Figure 2G). This demonstrates that both sounds and imagery cues induced similar activity patterns in early visual cortex and that feedback information is coded consistently across imagery and auditory perception. In auditory cortex, cross-classification did not succeed, indicating that activity patterns induced by feedforward auditory stimulation are coded differently than those induced by feedback through auditory imagery.

Decoding of Sounds while Manipulating Cognitive Resources

In experiments 3 and 4, we explored the robustness of cortical feedback to interference with orthogonal engagement of attention, working memory, and visuospatial processing.

[3]Present address: School of Psychology, University of East Anglia, Norwich Research Park, Norwich NR4 7TJ, UK
*Correspondence: petra.vetter@unige.ch (P.V.), lars.muckli@glasgow.ac.uk (L.M.)
This is an open access article under the CC BY license (http://creativecommons.org/licenses/by/3.0/).

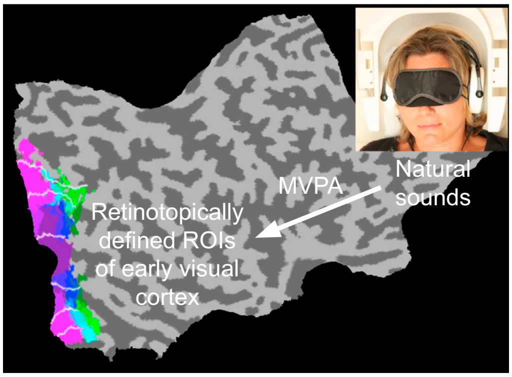

Figure 1. Experimental Setup and ROI Definition

In each of the five experiments, ten healthy subjects were scanned with solely auditory stimulation in the absence of visual stimulation. Subjects wore a blindfold and were instructed to keep their eyes closed at all times, and room lights were switched off. In a separate session, retinotopic mapping was performed for all subjects in all experiments to define early visual areas V1, V2, and V3. We show probability maps from the retinotopic mapping data of experiment 1 (n = 10) as derived from functionally informed cortex-based alignment on a flattened Montreal Neurological Institute (MNI) template. White lines indicate mean eccentricity boundaries. Sound-induced blood-oxygen-level-dependent activation patterns from these regions of interest (ROIs) were fed into a multivariate pattern analysis.

During natural sound stimulation, subjects performed an orthogonal task that was either an auditory working memory task (experiment 3) or a visuospatial imagery task (experiment 4). Again, both experiments omitted any visual stimulation. In experiment 3, subjects retained a list of five words (animals or everyday objects) in memory during the natural sound stimulation and subsequently matched it with a second word list in scrambled order (Figure 3A). Activity patterns during natural sound stimulation were again successfully decoded from early visual cortex, mainly in peripheral regions and consistently in V2 (Figures 3B and 3C). This demonstrates that simultaneous retention of orthogonal contents in working memory did not strongly affect classification.

In experiment 4, subjects engaged in an imaginary cube-assembly task [15]. Here, subjects mentally constructed an imaginary figure according to five assembly instructions and rotated the imaginary figure 90° clockwise while hearing the natural sound. Subsequently, they matched the rotated figure held in memory with a second list of instructions. Although the classifier failed to discriminate the three natural sounds in most of early visual cortex, residual above-chance classification remained in the far periphery of V2 (Figures 3E and 3F) despite the orthogonal engagement of attentionally demanding active visuospatial processing.

Whole-Brain Searchlight Results

We performed a whole-brain searchlight analysis to identify other areas that contain information from real and imagined sound content and may mediate information feedback to early visual cortex. Unsurprisingly, sounds could be decoded in a large part of bilateral superior temporal sulcus mostly belonging to auditory cortex (Figure 4). In experiments 1 and 2, real and imagined sounds could be decoded in parts of the precuneus and in posterior superior temporal sulcus (pSTS) (see overlapping regions in Figure 4). Sounds and, to a lesser extent, imagined sounds were successfully classified in a network of frontal regions, including superior and middle frontal sulci.

Univariate Activation Profile

Given previous controversial evidence of whether mental imagery elicits positive activity in early visual cortex, we performed a univariate generalized linear model analysis to see whether our decoding results were based on positive or negative activation profiles. Even at very liberal thresholds ($p < 0.05$ uncorrected; Figure S3), listening to sounds in the absence of visual stimulation elicited no positive activation in early visual areas but instead elicited a weak deactivation, consistent with previous findings (e.g.,[16]) and in contrast to classical findings for visual mental imagery [17, 18]. Imagery (experiment 2) elicited no positive activity but exhibited weak deactivations in both early visual and auditory cortices. In experiments 3 and 4, the secondary tasks activated early visual areas consistent with an engagement of object attention.

Category Specificity of the Information Fed Back to Early Visual Cortex

In experiment 5, we were interested in the specificity of the information that is fed back to early visual cortex. We hypothesized two possibilities: (1) sounds trigger a unique picture-like representation that reinstates the same activity patterns in early visual cortex as a real image does and thus allows successful decoding, and (2) higher-level abstract or categorical information is fed down to early visual cortex causing the differential activity patterns. The purpose of such information transfer could be to provide categorical expectations as proposed by models of predictive coding (e.g., [6, 7, 19]). We presented subjects with three different sound exemplars (6 s each) for each of the categories “human” and “inanimate.” The crucial experimental manipulation here was that two sound exemplars in each category could induce similar pictorial representations (different snapshots of a similar environment: “people 1” and “people 2” and “traffic 1” and “traffic 2”), whereas the third could induce a very different image due to a different feature (“playing kids” and “starting airplane”).

Classification of exemplars of the “human” versus the “inanimate” category was successful in several early visual areas for eight out of nine exemplar combinations (Figure 3G; Table S1), replicating in part the results of experiment 1 and demonstrating decoding of sounds of the categories “human” and “inanimate” with different sound exemplars and shorter stimulus presentation times.

Crucially, cross-classification succeeded in V2 and V3 in two out of three combinations, i.e., training the classifier for the pair “traffic 1” versus “people 1” lead to successful classification of “traffic 2” versus “people 2,” and training the classifier for the pair “traffic 2” versus “people 2” lead to successful classification of “airplane” versus “kids” (Figure 3H; Table S1). That is, the information contained in these activity patterns is generalizable across different sound exemplars within a category, demonstrating that sounds trigger shared categorical information transfer to early visual cortex rather than a fine-grained pictorial representation.

Discussion

Our series of five fMRI experiments provides converging evidence for consistent abstract information feedback from nonretinal sources to human early visual cortex.

We show that category-specific information from audition and imagery can be decoded from early visual cortex activity. The fact that our classifier could predict which sound was heard or imagined means that our results go beyond previous

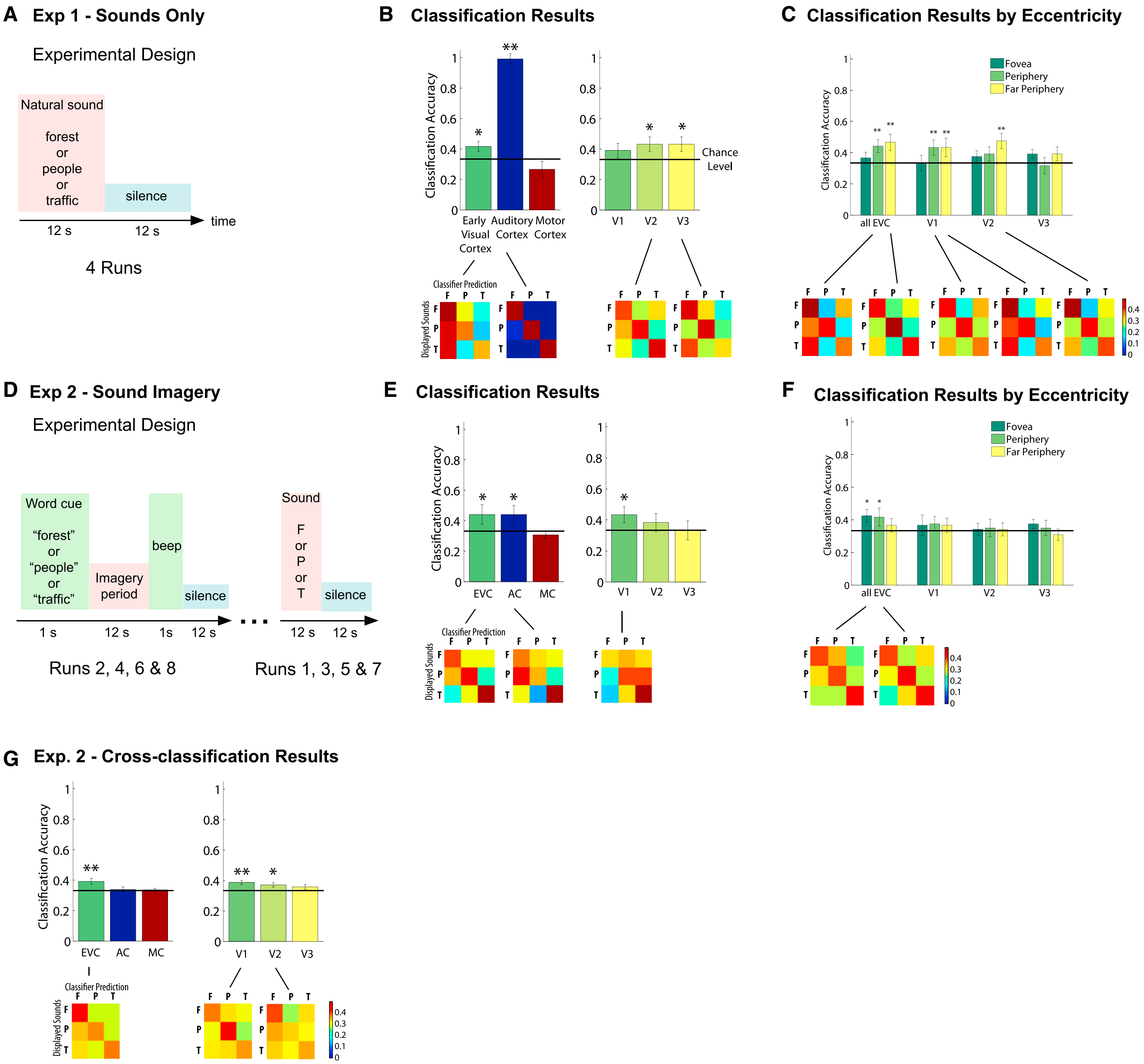

Figure 2. Experimental Design and Classification Results of Experiments 1 and 2

(A) In experiment 1, subjects listened to one of three different natural sounds, interleaved with silence (apart from scanner noise).

(B) Mean classification accuracy of the classifier distinguishing the three natural sounds in the different ROIs. Early visual cortex (EVC) contains V1, ventral V2, dorsal V2, ventral V3, and dorsal V3. Chance level is at one out of three. Error bars indicate SEM. All p values were derived from a permutation analysis (see Supplemental Experimental Procedures). Results for V1, V2, and V3 are single threshold corrected. $*p < 0.05$, $**p = 0.001$. For significant results, confusion matrices are displayed underneath the graphs to show that classification was not solely successful due to the difference between the activity patterns evoked by one sound versus all other patterns. Columns of the confusion matrices indicate the sound displayed (F, forest; p, people; T, traffic), and rows indicate which sound the classifier predicted. Classifier performance is represented by color hues, with warm colors for above-chance classification and cold colors for below-chance classification.

(C) Mean classification accuracies for all visual ROIs divided into three eccentricities (fovea, periphery, and far periphery). $*p < 0.05$ (uncorrected), $**p < 0.05$ (false discovery rate corrected).

(D) In experiment 2, subjects received a word cue to imagine the sounds and the associated visual scene. Four runs with word cues were alternated with four runs of actual sound stimulation.

(E) Classification results are shown for imagined sounds. $*p < 0.05$, $**p = 0.001$.

(F) Mean classification accuracies for different eccentricities of the visual ROIs. $*p < 0.05$ (uncorrected), $**p < 0.05$ (false discovery rate corrected).

(G) Cross-classification results of experiment 2. The classifier was trained on real sounds and tested on imagined sounds and vice versa, and results were averaged. $*p < 0.05$, $**p = 0.001$.

studies demonstrating an overall activity increase in early visual cortex in response to auditory stimulation [20] or visual mental imagery [17, 18]. Our study shows that sound stimulation and associated imagery generate shared and meaningful information feedback to early visual cortex, carrying abstract and possibly semantic information.

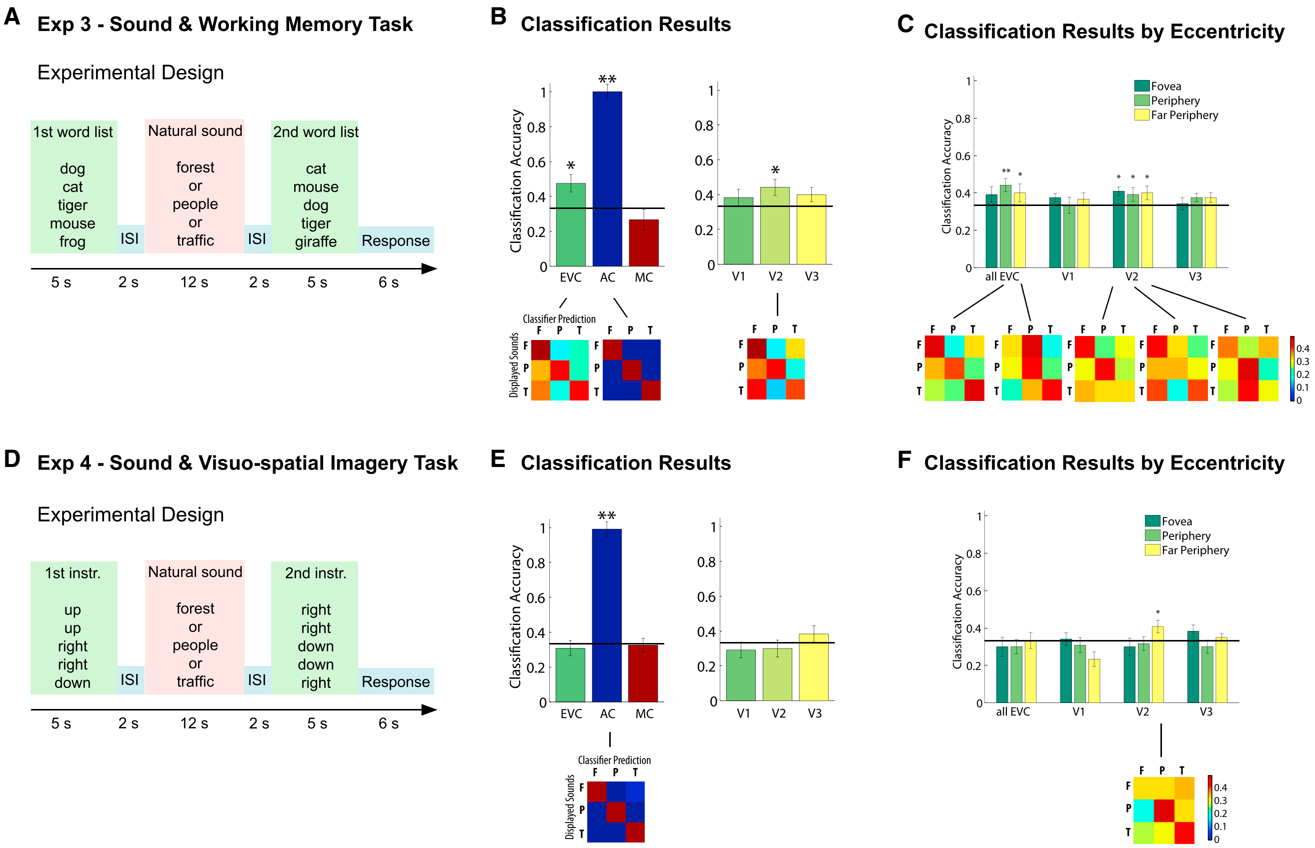

G Exp 5 - Classification Results

	Traffic 1	Traffic 2	Airplane
**People 1**	V2 & V3*	--	V2*
**People 2**	V1 ***<br>EVC*	V3d*<br>V2&V3*	V1*<br>V3v*<br>EVC**
**Kids**	V3 ***	V1*<br>V2*<br>V3***<br>V2&V3*<br>EVC**	V3d***<br>V3**

H Cross-classification Results

**Cross-classification**			
People 1 vs Traffic 1	X	People 2 vs Traffic 2	V2&V3*
People 2 vs Traffic 2	X	Kids vs Airplane	V2*<br>V2&V3*
People 1 vs Traffic 1	X	Kids vs Airplane	--

Figure 3. Experimental Design of Experiments 3 and 4 and Classification Results of Experiments 3, 4, and 5

(A) In experiment 3, subjects performed an orthogonal auditory working memory task while hearing natural sounds. They retained a word list of five animals or everyday objects in working memory and matched them with a second scrambled word list containing one different word in half of the trials. Match or mismatch was indicated with a button press during response time.

(B) Classification results for the three different sounds during performance of the task. Significance levels and analysis parameters were the same as in experiments 1 and 2. Error bars indicate SEM.

(C) Mean classification accuracies for all visual ROIs divided into three eccentricities (fovea, periphery, and far periphery).

(D) In experiment 4, subjects performed a visuospatial imaginary cube-assembly task while hearing natural sounds. They mentally constructed an imaginary figure according to five assembly instructions, rotated the imaginary figure 90° clockwise, and indicated match or mismatch of the correct solution with the second list of instructions.

(E) Classification results. *$p < 0.05$, **$p = 0.001$.

(F) Classification results by eccentricity of visual ROIs. *$p < 0.05$ (uncorrected), **$p < 0.05$ (false discovery rate corrected).

(G) In experiment 5, subjects listened to three different sound exemplars for each of the two categories, “human” (People 1, People 2, Kids) and “inanimate” (Traffic 1, Traffic 2, Airplane). Sounds were cut to 6 s, and interstimulus intervals were 6 s, otherwise the experimental design was the same as in experiment 1. The table shows early visual areas with significant above-chance classification for all combinations of “human” versus “inanimate” sounds. All p values were derived from permutation analyses. *$p < .05$, **$p < 0.005$, ***$p = 0.001$.

(H) Cross-classification of one pair of exemplars against another.

Previous studies focused on the decoding of visual mental imagery and the consistency of activity patterns across visual mental imagery and visual perception. Mostly, decoding of object categories worked in higher visual areas such as lateral occipital complex [21] or ventral temporal cortex [22] and to some extent in extrastriate cortex, but not in V1 [23, 24]. Our study is the first to show that inducing multisensory imagery allows decoding of complex mental imagery content in V1. Furthermore, whereas previous studies reported successful cross-classification between imagery and active visual

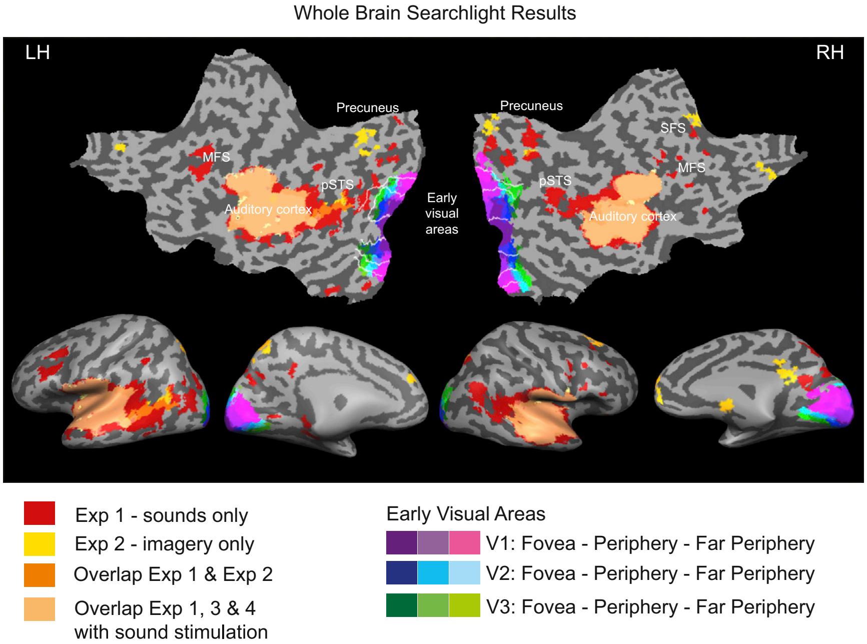

Figure 4. Results of the Whole-Brain Searchlight Analysis for Experiments 1–4

Overlay of significant above-chance classification of the three heard or imagined sounds onto a flattened and inflated cortical surface reconstruction (MNI template) for experiments 1–4. Note that a searchlight analysis is less sensitive than an ROI analysis because (1) the searchlight volume is small, and, thus, the classifier is less able to pick out subtle differences in activity patterns and because (2) correction for multiple comparisons is necessary on the whole-brain level (see Supplemental Experimental Procedures). Significance level is $p < 0.05$ with cluster threshold correction. Searchlight size was 343 voxels. For results with increased statistical power and a bigger searchlight, see Figure S4. Early visual areas depict probability maps as in Figure 1. pSTS, posterior superior temporal sulcus; SFS, superior frontal sulcus; MFS, middle frontal sulcus.

perception, our cross-classification analysis demonstrates a consistency of activity patterns in early visual areas across imagery and auditory perception. This is converging evidence that nonretinal feedback is consistent with respect to its semantic content, no matter its exact source.

Our results also show that this feedback is robust to mild interference with low attentional and working memory load (experiment 3) and to some extent even to interference with a visuospatially and attentionally highly demanding task (experiment 4).

The whole-brain searchlight analysis identified higher-level multisensory brain areas such as pSTS and precuneus possibly mediating the information feedback from sounds and imagery to early visual areas. The precuneus has been identified as an area responding to both visual and auditory stimuli and possibly serving as an audiovisual convergence area [25]. pSTS is implicated in audiovisual integration and has been shown to feed down information to primary visual and auditory cortices [26]. In the context of our findings, we suggest that the content-specific information from sounds, when they are heard and/or imagined, is relayed from auditory cortex to early visual cortex via pSTS and precuneus, eliciting differential activity patterns in both of these regions. Apart from the route via multisensory areas, there is evidence for multisensory integration on the subcortical level [27] and for direct anatomical connections between early auditory and early visual areas [12, 28, 29], mostly reaching peripheral regions [12–14], consistent with both our eccentricity and searchlight results. Also, hippocampal projections to peripheral early visual regions have been demonstrated in the context of boundary extension for scene processing [30]. However, whether these pathways play a causal role in inducing differential activity patterns remains to be investigated.

The successful classification in experiments 1 and 2 was driven by differential patterns of deactivation rather than activation, and, thus, our results are unlikely to be caused by the same neural mechanisms as those suggested in earlier studies on visual mental imagery [17, 18]. This also means that our results were not caused by an unspecific attention effect or a simple reactivation of early visual cortex due to pictorial visual mental imagery. The univariate activity profile also showed that classification was not driven by one sound eliciting more attention-related activity than another sound (Figure S3).

The results of experiment 5 suggest that the information that is fed down to early visual cortex is not only content specific but also category specific, i.e., related to the information shared by sound exemplars of the same category. This suggests that information feedback is unlikely to be caused by an exact pictorial representation but instead contains abstract and possibly semantic information. The findings of experiment 5 furthermore demonstrate that the successful decoding in experiment 1 was not specific to the first sound exemplars we used and could not be caused by differential low-level acoustic features of the sounds (e.g., frequency distribution).

Note that despite relatively low classification accuracies, our series of experiments replicated the successful decoding of sounds in early visual areas several times, demonstrating proof of principle and the robustness of our results across different subject and stimulus samples.

Previous fMRI studies using MVPA have provided evidence for nonfeedforward input to early visual cortex. For example, activity patterns in nonstimulated parts of early visual cortex contain content-specific information from the surrounding visual context [31, 32], from objects presented in the periphery [33], and from visual stimuli solely held in working memory rather than being actively perceived [34, 35]. Moreover, higher visual areas project back to V1 the associated color of grayscale objects [36] or the predicted motion path of an apparent motion illusion [37, 38]. Our results provide further novel evidence that early visual cortex receives category-specific feedback from auditory, multisensory, memory, or imagery areas in the absence of any actual visual stimulation. Furthermore, many studies of top-down or multisensory influences on sensory regions, such as the decoding of sound-implying visual images in auditory cortex [39], the decoding of touch-implying visual images in somatosensory cortex [40, 41], the recruitment of early visual cortex in blindfolded subjects by touch [42], or the decoding of memory traces in early visual cortex

[34, 35], could have been caused or accompanied by a form of mental imagery. Our study has explored the role of mental imagery in depth and has demonstrated that, in terms of reactivation of early visual cortex by a pictorial representation similar to actual visual perception, a simplistic mental imagery account falls short of explaining our results entirely.

Why should category-specific information be fed down all the way to early visual areas? One interpretation is that the brain provides priors fitting to the best prediction, and these priors can be transmitted between different sensory modalities. Within the framework of predictive coding, early sensory areas are "prepared" with a predictive model for the external incoming information through cortical feedback from higher cognitive areas, the hippocampus, and other sensory modalities [6–10, 43]. In the present case, early visual cortex may anticipate certain visual information due to real or imagined auditory information. That is, auditory stimulation or imagery triggers a predictive model reaching early visual areas via feedback connections from higher multisensory or imagery areas and evoking content-specific activity patterns. Our results demonstrate that the information arriving in early visual cortex is categorical and independent of its exact source. In fact, previous accounts suggested that prediction and mental imagery may involve overlapping brain mechanisms [6, 43, 44], and mental imagery might have evolved from predictive brain mechanisms. What distinguishes both from each other remains an interesting question to be investigated, both experimentally and theoretically. Omitting feedforward stimulation is a promising step in studying nonvisual input to early visual cortex; however, without feedforward stimulation, it is difficult to study the functional role of this influence in actual visual perception. Audiovisual priming studies with natural stimuli indicate a facilitatory role for visual perception [45].

Our results demonstrate that abstract information from nonretinal input, induced by both complex sound stimulation and mental imagery, can be translated to the coding space of early visual cortex. The purpose of such abstract information feedback might be to provide early visual cortex with a categorical prediction for the incoming visual input.

Supplemental Information

Supplemental Information includes Supplemental Experimental Procedures, three figures, and one table and can be found with this article online at http://dx.doi.org/10.1016/j.cub.2014.04.020.

Acknowledgments

This study was approved by the ethics committee of the College of Science and Engineering, University of Glasgow. This study was supported by BBSRC grant BB/G005044/1 and by ERC grant StG 2012_311751-BrainReadFBPredCode. We thank Lucy S. Petro, Frances Crabbe, Matt Bennett, Bahador Bahrami, Luca Vizioli, Philippe Schyns, Gregor Thut, and Wolf Singer.

Received: November 29, 2013
Revised: February 28, 2014
Accepted: April 8, 2014
Published: May 22, 2014

References

1. Spillmann, L. (2009). Phenomenology and neurophysiological correlations: two approaches to perception research. Vision Res. *49*, 1507–1521.
2. Salin, P.A., and Bullier, J. (1995). Corticocortical connections in the visual system: structure and function. Physiol. Rev. *75*, 107–154.
3. Markov, N.T., Vezoli, J., Chameau, P., Falchier, A., Quilodran, R., Huissoud, C., Lamy, C., Misery, P., Giroud, P., Ullman, S., et al. (2014). Anatomy of hierarchy: feedforward and feedback pathways in macaque visual cortex. J. Comp. Neurol. *522*, 225–259.
4. Self, M.W., van Kerkoerle, T., Supèr, H., and Roelfsema, P.R. (2013). Distinct roles of the cortical layers of area V1 in figure-ground segregation. Curr. Biol. *23*, 2121–2129.
5. Singer, W. (2013). Cortical dynamics revisited. Trends Cogn. Sci. *17*, 616–626.
6. Mumford, D. (1992). On the computational architecture of the neocortex. II. The role of cortico-cortical loops. Biol. Cybern. *66*, 241–251.
7. Friston, K. (2010). The free-energy principle: a unified brain theory? Nat. Rev. Neurosci. *11*, 127–138.
8. Clark, A. (2013). Whatever next? Predictive brains, situated agents, and the future of cognitive science. Behav. Brain Sci. *36*, 181–204.
9. Bar, M. (2007). The proactive brain: using analogies and associations to generate predictions. Trends Cogn. Sci. *11*, 280–289.
10. Bastos, A.M., Usrey, W.M., Adams, R.A., Mangun, G.R., Fries, P., and Friston, K.J. (2012). Canonical microcircuits for predictive coding. Neuron *76*, 695–711.
11. Muckli, L., Naumer, M.J., and Singer, W. (2009). Bilateral visual field maps in a patient with only one hemisphere. Proc. Natl. Acad. Sci. USA *106*, 13034–13039.
12. Eckert, M.A., Kamdar, N.V., Chang, C.E., Beckmann, C.F., Greicius, M.D., and Menon, V. (2008). A cross-modal system linking primary auditory and visual cortices: evidence from intrinsic fMRI connectivity analysis. Hum. Brain Mapp. *29*, 848–857.
13. Rockland, K.S., and Ojima, H. (2003). Multisensory convergence in calcarine visual areas in macaque monkey. Int. J. Psychophysiol. *50*, 19–26.
14. Cate, A.D., Herron, T.J., Yund, E.W., Stecker, G.C., Rinne, T., Kang, X., Petkov, C.I., Disbrow, E.A., and Woods, D.L. (2009). Auditory attention activates peripheral visual cortex. PLoS ONE *4*, e4645.
15. Sack, A.T., Jacobs, C., De Martino, F., Staeren, N., Goebel, R., and Formisano, E. (2008). Dynamic premotor-to-parietal interactions during spatial imagery. J. Neurosci. *28*, 8417–8429.
16. Laurienti, P.J., Burdette, J.H., Wallace, M.T., Yen, Y.F., Field, A.S., and Stein, B.E. (2002). Deactivation of sensory-specific cortex by cross-modal stimuli. J. Cogn. Neurosci. *14*, 420–429.
17. Slotnick, S.D., Thompson, W.L., and Kosslyn, S.M. (2005). Visual mental imagery induces retinotopically organized activation of early visual areas. Cereb. Cortex *15*, 1570–1583.
18. Amedi, A., Malach, R., and Pascual-Leone, A. (2005). Negative BOLD differentiates visual imagery and perception. Neuron *48*, 859–872.
19. Muckli, L., and Petro, L.S. (2013). Network interactions: non-geniculate input to V1. Curr. Opin. Neurobiol. *23*, 195–201.
20. Martuzzi, R., Murray, M.M., Michel, C.M., Thiran, J.P., Maeder, P.P., Clarke, S., and Meuli, R.A. (2007). Multisensory interactions within human primary cortices revealed by BOLD dynamics. Cereb. Cortex *17*, 1672–1679.
21. Stokes, M., Thompson, R., Cusack, R., and Duncan, J. (2009). Top-down activation of shape-specific population codes in visual cortex during mental imagery. J. Neurosci. *29*, 1565–1572.
22. Reddy, L., Tsuchiya, N., and Serre, T. (2010). Reading the mind's eye: decoding category information during mental imagery. Neuroimage *50*, 818–825.
23. Lee, S.-H., Kravitz, D.J., and Baker, C.I. (2012). Disentangling visual imagery and perception of real-world objects. Neuroimage *59*, 4064–4073.
24. Cichy, R.M., Heinzle, J., and Haynes, J.-D. (2012). Imagery and perception share cortical representations of content and location. Cereb. Cortex *22*, 372–380.
25. Hertz, U., and Amedi, A. (2010). Disentangling unisensory and multisensory components in audiovisual integration using a novel multifrequency fMRI spectral analysis. Neuroimage *52*, 617–632.
26. Naumer, M.J., van den Bosch, J.J.F., Wibral, M., Kohler, A., Singer, W., Kaiser, J., van de Ven, V., and Muckli, L. (2011). Investigating human audio-visual object perception with a combination of hypothesis-generating and hypothesis-testing fMRI analysis tools. Exp. Brain Res. *213*, 309–320.
27. van den Brink, R.L., Cohen, M.X., van der Burg, E., Talsma, D., Vissers, M.E., and Slagter, H.A. (2013). Subcortical, modality-specific pathways contribute to multisensory processing in humans. Cereb. Cortex. Published online March 25, 2013. http://dx.doi.org/10.1093/cercor/bht069.

28. Beer, A.L., Plank, T., Meyer, G., and Greenlee, M.W. (2013). Combined diffusion-weighted and functional magnetic resonance imaging reveals a temporal-occipital network involved in auditory-visual object processing. Front Integr Neurosci *7*, 5.
29. Beer, A.L., Plank, T., and Greenlee, M.W. (2011). Diffusion tensor imaging shows white matter tracts between human auditory and visual cortex. Exp. Brain Res. *213*, 299–308.
30. Chadwick, M.J., Mullally, S.L., and Maguire, E.A. (2013). The hippocampus extrapolates beyond the view in scenes: an fMRI study of boundary extension. Cortex *49*, 2067–2079.
31. Smith, F.W., and Muckli, L. (2010). Nonstimulated early visual areas carry information about surrounding context. Proc. Natl. Acad. Sci. USA *107*, 20099–20103.
32. Ban, H., Yamamoto, H., Hanakawa, T., Urayama, S.-I., Aso, T., Fukuyama, H., and Ejima, Y. (2013). Topographic representation of an occluded object and the effects of spatiotemporal context in human early visual areas. J. Neurosci. *33*, 16992–17007.
33. Williams, M.A., Baker, C.I., Op de Beeck, H.P., Shim, W.M., Dang, S., Triantafyllou, C., and Kanwisher, N. (2008). Feedback of visual object information to foveal retinotopic cortex. Nat. Neurosci. *11*, 1439–1445.
34. Harrison, S.A., and Tong, F. (2009). Decoding reveals the contents of visual working memory in early visual areas. Nature *458*, 632–635.
35. Albers, A.M., Kok, P., Toni, I., Dijkerman, H.C., and de Lange, F.P. (2013). Shared representations for working memory and mental imagery in early visual cortex. Curr. Biol. *23*, 1427–1431.
36. Bannert, M.M., and Bartels, A. (2013). Decoding the yellow of a gray banana. Curr. Biol. *23*, 2268–2272.
37. Muckli, L., Kohler, A., Kriegeskorte, N., and Singer, W. (2005). Primary visual cortex activity along the apparent-motion trace reflects illusory perception. PLoS Biol. *3*, e265.
38. Vetter, P., Grosbras, M.-H., and Muckli, L. (2013). TMS over V5 disrupts motion prediction. Cereb. Cortex. Published online October 23, 2013. http://dx.doi.org/10.1093/cercor/bht297.
39. Meyer, K., Kaplan, J.T., Essex, R., Webber, C., Damasio, H., and Damasio, A. (2010). Predicting visual stimuli on the basis of activity in auditory cortices. Nat. Neurosci. *13*, 667–668.
40. Smith, F.W., and Goodale, M.A. (2013). Decoding visual object categories in early somatosensory cortex. Cereb. Cortex. Published online October 11, 2013. http://dx.doi.org/10.1093/cercor/bht292.
41. Meyer, K., Kaplan, J.T., Essex, R., Damasio, H., and Damasio, A. (2011). Seeing touch is correlated with content-specific activity in primary somatosensory cortex. Cereb. Cortex *21*, 2113–2121.
42. Merabet, L.B., Hamilton, R., Schlaug, G., Swisher, J.D., Kiriakopoulos, E.T., Pitskel, N.B., Kauffman, T., and Pascual-Leone, A. (2008). Rapid and reversible recruitment of early visual cortex for touch. PLoS ONE *3*, e3046.
43. Maguire, E.A., and Mullally, S.L. (2013). The hippocampus: a manifesto for change. J. Exp. Psychol. Gen. *142*, 1180–1189.
44. Moulton, S.T., and Kosslyn, S.M. (2009). Imagining predictions: mental imagery as mental emulation. Philos. Trans. R. Soc. Lond. B Biol. Sci. *364*, 1273–1280.
45. Chen, Y.-C., and Spence, C. (2010). When hearing the bark helps to identify the dog: semantically-congruent sounds modulate the identification of masked pictures. Cognition *114*, 389–404.

Current Biology, Volume *24*

Supplemental Information

Decoding Sound and Imagery Content

in Early Visual Cortex

Petra Vetter, Fraser W. Smith, and Lars Muckli

Supplemental Material

Supplemental Results

Experiments 1 - 4

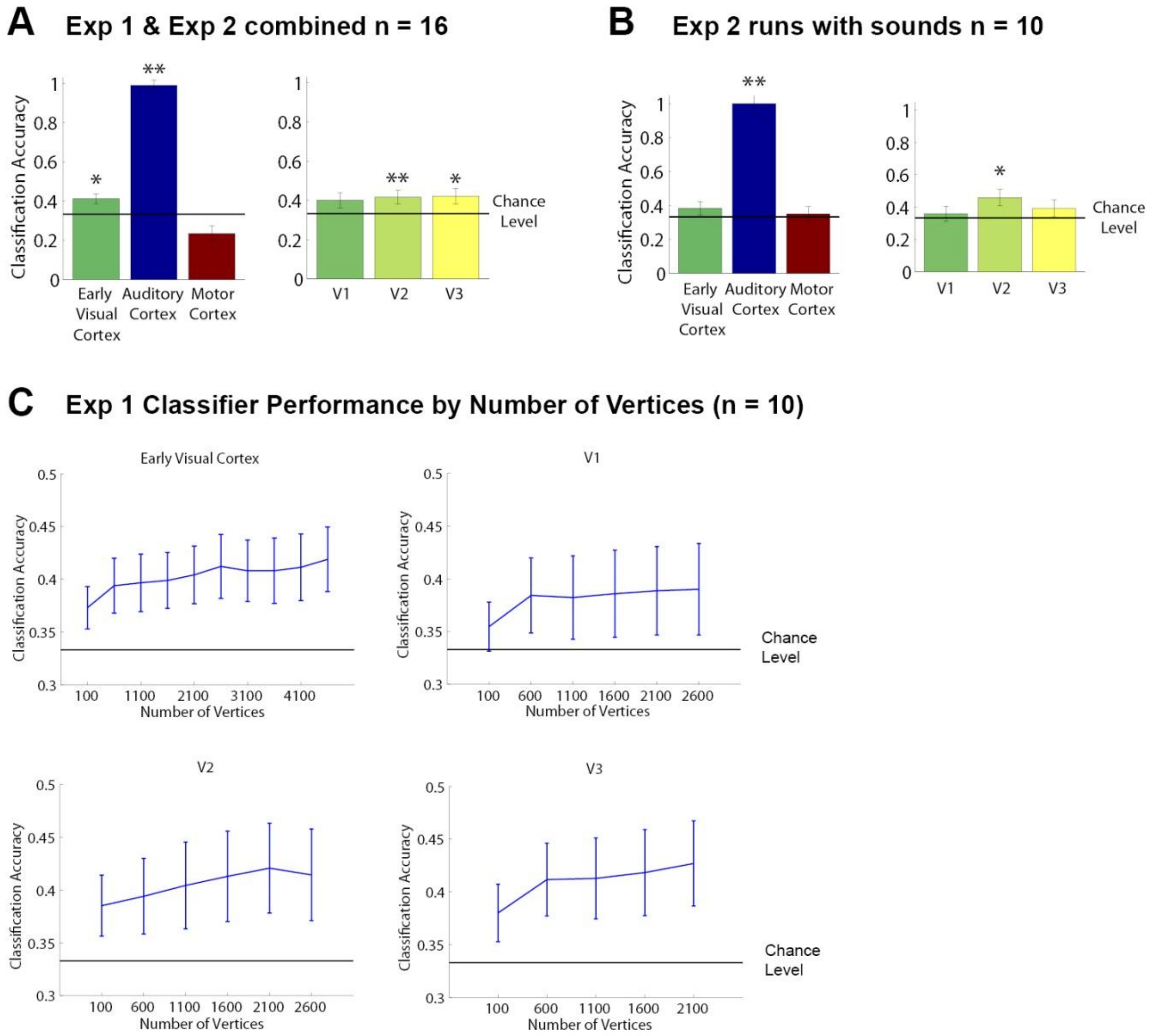

Figure S1. Classification results from Exp. 1 & 2. A) Results from the pooled data set with higher statistical power (n = 16). Here the data of Exp. 1 was pooled with the data of the runs with sound stimulation in Exp. 2, excluding the subjects who participated in both experiments (n = 4). B) Results from Exp. 2 for the runs with sound stimulation, replicating the results of Exp. 1. C) Classification performance in Exp. 1 as a function of number of vertices in each visual ROI. All error bars indicate SEM.

Effect sizes ((mean decoding accuracy-chance)/ STD) for the pooled data set were as follows: Early visual cortex: 0.79; V2: 0.60; V3: 0.57; Auditory cortex: 23.0.

Comparison of confusion matrices between Exp. 1 and Exp. 3 for V3 and early visual cortex revealed no significant difference (repeated measures ANOVA, $p = .60$) and a significant correlation in early visual cortex (Spearman's $r = .726$; $p = .027$). That is, successful sound decoding in both experiments relied on similar patterns of classifier performance.

Repeated measures ANOVA for classification accuracies across the early visual areas V1, V2 and V3 revealed non-significant effects of visual area ($F(2,18) < 1.7$, $p > .20$) in all experiments. Therefore, the data does not allow us to conclude a differential involvement of individual early visual areas across the different experiments.

Behavioural task performance (mean accuracy) in Exp. 3 & Exp. 4:

Exp. 3: 90.4% (SEM 2.9); Exp. 4: 90% (SEM .02).

Whole-brain Searchlight Results

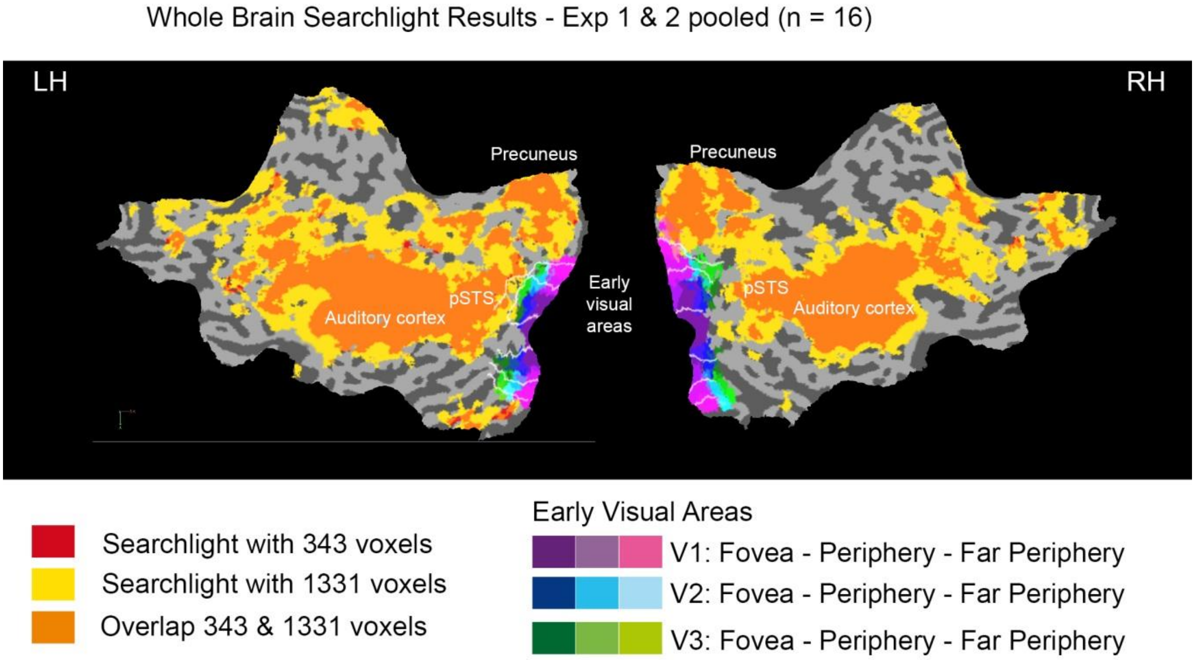

Figure S2. Searchlight results for the pooled data set (Exp. 1 & 2, n = 16) for two different searchlight sizes (a cube of 343 voxels and a cube of 1331 voxels).

Univariate Results

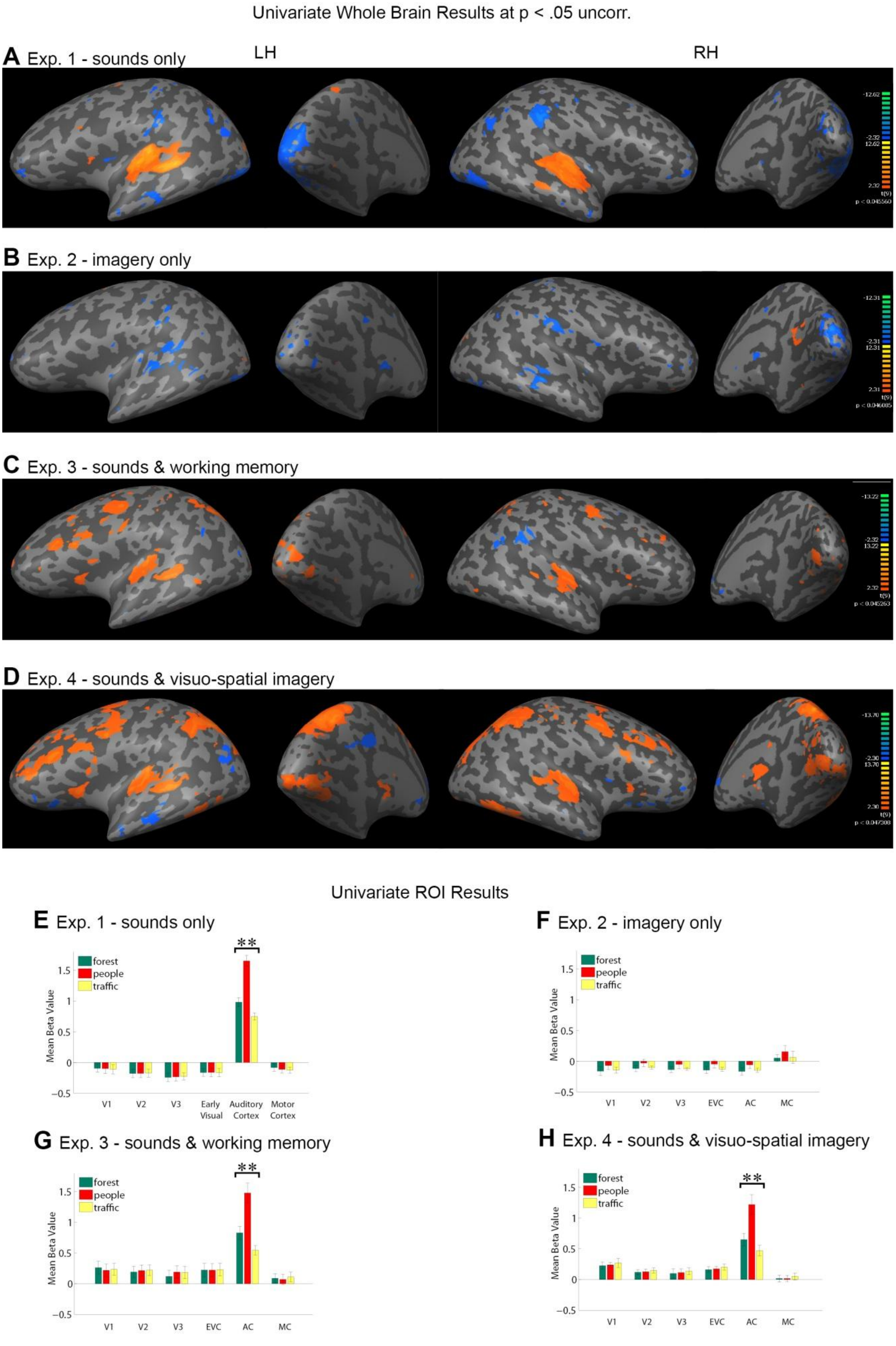

Fig. S3. Results of the univariate analysis for Exp. 1- 4.

A) - D) Whole-brain results from a random effects analysis projected onto cortical surfaces (MNI template) for Exp. 1- 4 at $p < .05$ uncorr. E) - H) Mean beta values for the three heard or imagined sounds in the relevant ROIs. Error bars indicate SEM. ** indicate $p <.001$ for a differential effect of sounds.

Sound display activated auditory cortex in Exp. 1, 3 & 4 and the orthogonal tasks of Exp. 3 and 4 activated a network of parietal and frontal regions due to the task demands (stronger activity for Exp. 4, particularly in parietal regions typically related to visuo-spatial processing). Note that even at very liberal thresholds of $p >.05$ (uncorrected), no positive activation was found in early visual areas, instead weak deactivation in Exp. 1 and 2.

Classification Results of Experiment 5

All classifications between categories and all cross-classifications were highly successful in auditory cortex ($p =. 001$).

	**Traffic 1**	**Traffic 2**	**Airplane**
**People 1**	V2 & V3* (.542 /.021)	--	V2* (.532 / .019)
**People 2**	V1 *** (.553 / .020)<br>EVC* (.532 / .026)	V3d * (.543 / .032)<br>V2&V3* (.535 / .030)	V1* (.537 / .017)<br>V3v* (.547 / .020)<br>EVC** (.565 / .023)
**Kids**	V3 *** (.555 / .017)	V1* (.550 / .026)<br>V2* (.528 / .037)<br>V3*** (.578 / .041)<br>V2&V3* (.538 / .040)<br>EVC** (.550 / .032)	V3d*** (.560 / .020)<br>V3** (.558 / .032)

**People 1 vs Traffic 1 X People 2 vs Traffic 2**	V2&V3* (.524 / .014)
**People 2 vs Traffic 2 X Kids vs Airplane**	V2* (.531 / .016)<br><br>V2&V3* (.523 / .013)
**People 1 vs Traffic 1 X Kids vs Airplane**	--

Supplemental Table 1. Classification Accuracies of Exp. 5.

Early visual areas with significant above chance classification for all combinations of "human" versus "inanimate" sounds, and cross-classification between one pair of exemplars against another, shown with mean classification accuracy and SEM (acc / SEM). All p - values were derived from permutation analyses. *p < . 05, ** p < .005, *** p = .001.

In theory, there is the possibility that differential eye movements in response to the sounds might have caused the differential activity patterns in early visual areas. We have no indication to believe that subjects moved their eyes systematically while being blindfolded and even if they did, activity patterns should not have been caused by retinal stimulation. Furthermore, two pieces of evidence in our findings speak against this possibility. First, the searchlight analysis did not yield successful classification in frontal eye fields. Second, Exp. 5 showed that sound decoding is based on categorical information, and it is not plausible to assume that eye movement patterns should follow this categorical distinction.

Supplemental Experimental Procedures

Subjects, stimuli and experimental design

10 healthy subjects with normal hearing and vision were scanned in each of the five experiments. Subjects signed informed consent. The study was approved by the ethics committee of the College of Science and Engineering, University of Glasgow. Stimulation was solely auditory through noise-reducing headphones (Nordic NeuroLab); subjects wore a blindfold, were instructed to keep their eyes closed at all times and room lights were switched off. The three natural sound stimuli used in Exp. 1- 4 consisted of one exemplar each of traffic noise (a busy road with cars and motorbikes), a forest scene (birds singing and a stream) and a crowd scene (people talking without clear semantic information) and were downloaded from www.soundsnap.com and cut to 12s. In Exp. 5, in addition to the traffic and people sound from experiments 1, 3 & 4, sounds of another traffic scene, a starting airplane, another crowd scene and playing children were used. Here, sounds were cut to 6s. In all experiments, sounds were normalised for amplitude and presented mono.

Natural sounds (Exp. 1, 3, 4 & 5) or a 12s imagery period (Exp. 2) were repeated 6 times per run (pseudo-randomised, but never repeating two of the same after the other). For timings and experimental design, see Fig. 2 & 3. In Exp. 3 & 4, subjects indicated match or mismatch with a right hand button press on a response pad. 4 runs (222 volumes each) were recorded in Exp. 1-4, 5 runs (225 volumes each) in Exp. 5. In Experiment 2, a replication of Experiment 1 was incorporated such that runs with real sound stimulation alternated with runs with imagery cues (4 runs of each type, 8 runs in total).

fMRI data acquisition and analysis

Blood oxygen level dependent signals were acquired in a 3 T Siemens Tim Trio (TR = 2s, TE = 30 ms, resolution 2.5 x 2.5 x 2.5 mm, 35 slices, flip angle 77º, iPAT factor 2). Early visual areas were identified in each individual subject using standard retinotopic polar mapping [13; S1-S2]. In Exp. 1, 3, 4 & 5, auditory cortex was identified as the area in superior temporal sulcus with peak activation for the contrast Sound Stimulation > Baseline. In Exp. 2, auditory cortex was identified from the interleaved runs with sound stimulation. In Exp. 3 & 4, motor cortex was defined in only the left hemisphere as the peak activation for the contrast Right Hand Button Press > Baseline. In Exp. 1 & 2, motor cortex was defined by overlaying the averaged group-level peak activation of Exp. 3 or 4 onto the individual brains of those subjects who did not participate in either Exp. 3 or 4. Data were analysed with BrainVoyager QX (BrainInnovation) with standard preprocessing (including slice scan time correction, no spatial smoothing, temporal high-pass filter, 3D rigid body motion correction). Regions of interest (ROIs) were defined on individual reconstructed cortical surfaces and based on retinotopic mapping. Single block beta weights were estimated for all vertices of each ROI during natural sound stimulation or imagery period [38] and fed into a linear support vector machine classification algorithm (LIBSVM toolbox [S3]). Beta values were normalised in the training data set and the same normalisation was applied for the testing data. The classification was performed one-versus-one for each of the three combinations of sounds and results were averaged. ROIs were combined across both hemispheres, whereas for motor cortex, only the activity patterns of the left hemisphere were analysed (due to right-hand button press). Mean number of vertices across all subjects (combined hemispheres) were as follows: V1: 4908 (SEM 245), V2: 3503 (SEM 164), V3: 2792 (SEM 126),

all early visual cortex: 11236 (SEM 429), auditory cortex ROI: 3311 (SEM 346), motor cortex ROI: 535 (SEM 72)). In Exp. 1-4, the classifier was trained on 3 runs to distinguish between the three types of natural sounds and tested on the remaining 4th run in a leave-one-run-out cross-validation procedure (results were averaged across different folds of training and test data set assignments). For the cross-classification analysis of Exp. 2, the classifier was trained on the runs with sound stimulation and tested on the runs with imagery cues and vice versa, and the results averaged. In Exp. 5, the classifier was trained on 4 runs to distinguish between the two sound categories (human and inanimate) and tested on the remaining 5th run in the same cross-validation procedure. Here, the classification was performed for all 9 combinations of inanimate versus human sound exemplars and the cross-classification was performed for the 3 combinations of one pair of exemplars versus one of the other two pairs. Within each cross-classification, results from training one data set and testing the other and vice versa were averaged. To determine statistical significance, a permutation analysis was performed for all experiments and all classifications, providing a more robust test of statistical significance than a one-sample t-test against chance [S4]. Here, the classifier was trained and tested across 1000 permutations with randomised labels in each subject and each ROI. P values were derived as the probability of getting a value as large as the real label performance in the randomisation distribution, resulting in a smallest possible p-value of 0.001 [S5]. On the group level, p-values were derived from the mean randomisation distribution and the mean real label performance. In Exp. 1-4, p-values were corrected for multiple comparisons with a single threshold test [S5] for the individual visual areas V1, V2 and V3.

Whole brain searchlight analyses were performed on the voxel level with the

SearchMight toolbox [S6] using a linear SVM (with C=1). Each searchlight consisted of 343 voxels (a cube with 7 voxels length, equal to 2744 cubic mm). Statistical significance was assessed by testing whether the mean accuracy across participants was significantly higher than chance (1/3) at each voxel (see also [S7]). Results were corrected for multiple comparisons with a cluster threshold correction ($p < .05$) estimated by the BrainVoyager Cluster Threshold Plugin tool.

Supplemental References

S1. Wandell, B. A., Dumoulin, S. O., & Brewer, A. A. (2007).Visual field maps in human cortex. Neuron, 56(2), 366–383.

S2. Schira, M. M., Tyler, C. W., Breakspear, M., & Spehar, B. (2009). The foveal confluence in human visual cortex. J. Neurosci, 29(28), 9050–9058.

S3. Chang, C.C., Lin, C.J. (2001). http://www.csie.ntu.edu.tw/~cjlin/libsvm.

S4. Stelzer, J., Chen, Y., & Turner, R. (2013). Statistical inference and multiple testing correction in classification-based multi-voxel pattern analysis (MVPA): random permutations and cluster size control. NeuroImage, 65, 69–82.

S5. Nichols, T.E. & Holmes, A.P. (2002). Nonparametric permutation tests for functional neuroimaging: a primer with examples. Hum Brain Mapp, 15, 1-25.

S6. Pereira, F., & Botvinick, M. (2011). Information mapping with pattern classifiers: a comparative study. NeuroImage, 56 (2), 476–496.

S7. Walther, D. B., Caddigan, E., Fei-Fei, L., & Beck, D. M. (2009). Natural scene categories revealed in distributed patterns of activity in the human brain. J. Neurosci, 29(34), 10573–10581.
